# When research data meet reality: contrasting advanced laryngeal cancer outcomes in The Cancer Imaging Archive (TCIA) and a United Kingdom regional cohort

**DOI:** 10.1017/S0022215125103538

**Published:** 2026-01

**Authors:** Amar Rajgor, Christopher Kui, Aileen Mill, Stephen Rushton, Boguslaw Obara, David Winston Hamilton

**Affiliations:** 1 Newcastle University, Newcastle-Upon-Tyne, UK; 2 National Institute for Health Research, Newcastle University, Newcastle-Upon-Tyne, UK; 3 Newcastle-Upon-Tyne Hospitals NHS Foundation Trust, Freeman Hospital, Newcastle-Upon-Tyne, UK

**Keywords:** Laryngeal, Neoplasms, Prognosis, Head and Neck Cancer, survival outcomes, research inequalities

## Abstract

**Objectives:**

Clinical trials provide valuable treatment insights but often fail to represent real-world outcomes. This is particularly true for advanced laryngeal cancer patients, who face significant co-morbidities and socioeconomic challenges. This study evaluates whether outcomes from research datasets in The Cancer Imaging Archive reflect real-world survival in a regional cohort from North-East England.

**Methods:**

This retrospective analysis compares outcomes between The Cancer Imaging Archive (n = 198) and North-East England (n = 222) cohorts. Demographics, treatment modalities and five-year disease-specific survival were assessed via Kaplan–Meier curves and Cox regression.

**Results:**

North-East England had a lower five-year disease-specific survival (59.2 per cent vs. 76.9 per cent; *p* = 0.0018) and was characterised by greater co-morbidity burden and upfront surgery with adjuvant therapy (51 per cent), whereas The Cancer Imaging Archive patients received upfront chemoradiotherapy (53 per cent) or radiotherapy alone (41 per cent).

**Conclusion:**

The poorer real-world outcomes reflect the challenges of generalising research data to heterogeneous populations. Bridging the gap between research efficacy and real-world effectiveness is critical to delivering equitable care for advanced laryngeal cancer.

## Introduction

Approximately 2,400 cases of laryngeal cancer are diagnosed each year in the United Kingdom (UK), reflecting 10-20 per cent of all head and neck cancers (HNCs).^1^^,^^2^ In England, a substantial proportion of patients are diagnosed with early stage (T1) laryngeal cancer, which is associated with excellent five-year survival rates of approximately 90 per cent.^3^^–^^5^ However, nearly half of all patients present with more advanced disease (T3 or T4),^4^ where five-year disease-specific survival (DSS) drops significantly to around 50 per cent.^3^^,^^6^^–^^8^ For patients with advanced laryngeal cancer (ALC), treatment decisions are guided by multi-disciplinary teams (MDTs) and may include total laryngectomy, concurrent chemoradiotherapy, or radiotherapy alone, depending on patient co-morbidities, performance status and preferences regarding laryngeal preservation.^2^ The poor prognosis in ALC underscores a need for effective treatment strategies.

Clinical trials remain central to evaluating treatment efficacy. By design, they aim to minimise confounding through selective inclusion criteria, often enrolling fitter, more homogeneous patient populations. While this approach strengthens internal validity, it can limit external applicability, particularly in real-world settings where patients may have multiple co-morbidities and social challenges.^9^^–^^13^ This issue is especially relevant for ALC, where it is unclear whether trial-based outcomes, such as those reported in The Cancer Imaging Archive (TCIA),^14^ reflect those seen in unselected clinical populations.

TCIA is an open international repository of de-identified cancer imaging datasets that are often linked to clinicopathological variables and outcomes. Much of TCIA derives from clinical trials or carefully selected research cohorts, which typically enrol fitter patients with fewer co-morbidities and more complete treatment delivery. While this improves internal validity, it may reduce applicability to routine clinical practice, where patients often present with multiple co-morbidities, socioeconomic challenges and variable treatment tolerance. Our study therefore contrasts TCIA with a consecutive UK regional cohort to illustrate how research-derived results may differ in real-world settings.

The population of North-East England (NEE), United Kingdom, experiences significant health inequalities, high rates of co-morbidities and increased adverse health behaviours (smoking and alcohol use) relative to the remainder of England.^15^^,^^16^ This study aims to generate real-world data from a tertiary head and neck centre in NEE treating patients with ALC. By comparing baseline characteristics, treatment patterns and survival outcomes with those reported in TCIA research datasets, we assess the applicability of such findings to real-world populations and highlight key differences that may influence treatment decisions and outcomes.

## Methods

### North-East England (NEE) dataset

Our retrospective cohort comprises 222 patients with advanced laryngeal (T3/T4) squamous cell cancer (SCC), as defined by the eighth edition American Joint Committee on Cancer (AJCC) cancer staging system.^17^ The Newcastle-Upon-Tyne NHS Foundation Trust (United Kingdom) delivers a tertiary HNC service to the NEE region. Following institutional approval, data were collected from otorhinolaryngology clinics or HNC MDT meetings. Patients were included if they were discussed at the regional MDT with initial curative intent, even if subsequent assessment recognised that prognosis was poor and management became non-curative. No selective inclusion was applied. Demographic, clinicopathological and outcomes data were examined, with a median follow-up period of 27 months. Of the NEE cohort, 13 per cent of patients received supportive care only. These patients were excluded from the survival analyses to allow consistent comparison with the research-based TCIA cohort. Their presence in the NEE dataset, however, reflects the reality of a consecutive regional cohort, in which patients with advanced disease but poor prognosis are still assessed at MDT, even if ultimately managed non-curatively.

### TCIA dataset

The Cancer Imaging Archive serves primarily as a cancer imaging repository and contains publicly accessible, de-identified medical data.^14^ Image datasets hosted on TCIA are often stored with relevant clinicopathological data. With approval from the TCIA, we curated a comparative cohort comprising 19 patients with advanced laryngeal (T3/T4) SCC. The data were collated from five research datasets available on the TCIA; Head-Neck-PET-CT (data from four centres in Quebec, Canada),^18^^,^^19^ Head-Neck-Radiomics-HN1 (single-centre data from the Maastro Clinic, Netherlands),^20^^,^^21^ HNSCC (single-centre data from MD Anderson, Texas, USA),^22^ QIN-HeadNeck (data collected by the University of Iowa, USA),^23^^,^^24^ TCGA-HNSC (data collected from five universities across the USA).^25^ A sixth TCIA dataset (HNSCC-3DCT-RT) was initially examined but excluded from analysis due to the lack of available survival data beyond three to six months.^26^ As the full datasets included other tumour types, strict selection criteria were applied to ensure meaningful comparison with the NEE cohort (Table 1). The final TCIA cohort had a median follow-up period of 37 months.

**Table 1.** TCIA cohort and selection criteria

**Dataset information**	TCIA dataset	Head-Neck-PET-CT	Head-Neck-Radiomics-HN1	HNSCC	QIN-HeadNeck	TCGA-HNSC
	Publication	Vallières et al. 2017	Aerts et al. 2014	N/A	Kalpathy-Cramer et al. 2014	N/A
	Region	Quebec, Canada	Netherlands	Texas, USA	Iowa, USA	USA
	Centres	Four	Single Maastro Clinic	Single MD Anderson	University of Iowa	Five
	Sample size	300	136	215	278	88,991
**Exclusion criteria**	Data error	2	0	0	0	0
	Non-laryngeal cancer	77	88	191	234	88,874
	Early (T1/T2) laryngeal cancer	1	24	1	17	25
	Insufficient clinical data	0	0	0	0	0
**Final cohort**	Advanced (T3/T4) laryngeal cancer with clinical data	32	24	23	27	92
	TCIA cohort	n = 198				

*Note:* Overview of The Cancer Imaging Archive (TCIA) datasets included in this study and the selection process yielding a final cohort of 198 advanced (T3/T4) laryngeal cancer patients.T4) squamous cell cancer.

Abbreviation: TCIA = The Cancer Imaging Archive.

### Statistical analysis

Statistical analysis was carried out using R version 4.4.3 and RStudio version 2024.12.1+563. The chi-squared and Student’s t-test were employed to examine differences in characteristics between the NEE and TCIA cohorts, with a *p* value less than 0.05 representing statistical significance. For both cohorts, we examined five-year DSS, overall survival (OS) and recurrence-free survival (RFS) via Kaplan–Meier analysis. DSS was defined as time from diagnosis to death attributed to laryngeal cancer. In UK practice, cancer is often recorded on death certificates even when not the direct cause. To reduce this potential misclassification, we evaluated electronic records and clinical notes for each patient, and attribution of cancer-specific death was made as a clinical decision rather than relying solely on the death certificate. Deaths clearly unrelated to cancer were censored at the date of death. While this approach reduces over-attribution, some residual misclassification is possible.

The impact of treatment modality on five-year DSS was explored via univariate Cox regression analysis, with calculation of both hazard ratios (HRs) and 95 per cent confidence intervals (CIs). Patients who received supportive care alone were excluded from all survival analyses to ensure comparability across treatment modalities and cohorts.

## Results

### Baseline characteristics

The NEE cohort comprised 222 patients with advanced laryngeal SCC. The TCIA cohort comprised 198 patients pooled across 5 research datasets (Table 1); Head-Neck-PET-CT (*n* = 32/198; 16 per cent),^18^^,^^19^ Head-Neck-Radiomics-HN1 (*n* = 24/198; 12 per cent),^20^^,^^21^ HNSCC (*n* = 23/198; 12 per cent),^22^ QIN-HeadNeck (*n* = 27/198; 14 per cent),^23^^,^^24^ TCGA-HNSC (*n* = 92/198; 46 per cent).^25^

The two cohorts had comparable baseline demographics, with no significant differences in age, gender, or ALC stage distribution (Table 2). The TCIA cohort had substantial missing data for smoking (29 per cent), alcohol intake (54 per cent), tumour subsite (80 per cent); the NEE cohort provided comprehensive data, reporting greater proportion of current smokers (51 per cent vs. 37 per cent TCIA).

**Table 2.** Baseline characteristics for the NEE and TCIA cohorts

Characteristics	NEE Cohort (n = 222)		TCIA Cohort (n = 198)		*p*-Value (X2)
Age (mean ± SD)	65.8	(± 12.3)	61.8	(± 9.2)	–
Gender					
Male	179	(81%)	166	(85%)	0.275
Female	43	(19%)	30	(15%)	
Smoking status					
Non-smoker	11	(5%)	22	(11%)	–
Ex-smoker	98	(44%)	45	(23%)	–
Current smoker	113	(51%)	73	(37%)	–
N/A	–	–	56	(29%)	–
Alcohol intake					
Below recommended units	134	(60%)	29	(15%)	–
Greater than recommended units	56	(25%)	61	(31%)	–
Previous excess	32	(14%)	–	–	–
N/A	–	–	106	(54%)	–
TNM status (TNM 8)					
T3	137	(62%)	118	(60%)	0.753
T4	85	(38%)	78	(40%)	
N0	116	(52%)	93	(47%)	0.327
N1	19	(9%)	26	(13%)	0.121
N2	65	(29%)	70	(36%)	0.160
N3	22	(10%)	7	(4%)	0.011*
M0	218	(98%)	193	(98%)	0.829
M1	4	(2%)	3	(2%)	
Tumour subsite					
Subglottic	4	(2%)	0	(0%)	–
Glottic	22	(10%)	8	(4%)	–
Transglottic	90	(48%)	–	–	–
Supraglottic	106	(41%)	31	(16%)	–
N/A	–	–	157	(80%)	–
Primary treatment					
Radiotherapy	26	(12%)	80	(41%)	< 0.001*
Chemoradiotherapy	54	(24%)	104	(53%)	< 0.001*
Surgery alone	46	(21%)	1	(1%)	< 0.001*
Surgery + adjuvant therapy	67	(30%)	11	(6%)	< 0.001*
Supportive care	29	(13%)	–	–	–

*Note:* Demographics and primary treatment in the NEE and TCIA cohorts; χ^2^ tests applied where complete data were available.

Abbreviations: NEE = North-East England; TCIA = The Cancer Imaging Archive.

### Treatment modality

Significant differences in upfront treatment modality were observed (*p* < 0.001) (Table 2). In the NEE cohort, 51 per cent of patients underwent surgical resection with or without adjuvant therapy, while 13 per cent received supportive care focused on symptom control and non-curative intent. In contrast, the TCIA cohort primarily received radiotherapy alone (41 per cent) or chemoradiotherapy (53 per cent), with no patients receiving supportive care—consistent with the research-focused nature of the cohort. To enable a consistent assessment of survival outcomes across treatment modalities with curative intent, patients receiving supportive care alone were excluded from survival comparisons.

### Survival outcomes

Patients who received supportive care alone were excluded from these comparisons to ensure consistent evaluation across curative-intent treatment modalities. Both cohorts comprised only advanced-stage laryngeal SCC (T3/T4), ensuring stage-appropriate comparisons. The distribution of T3 versus T4 disease did not differ significantly between NEE and TCIA (Table 2). Further stratification by T stage was considered but avoided, as it would have reduced statistical power.

**Figure 1.**
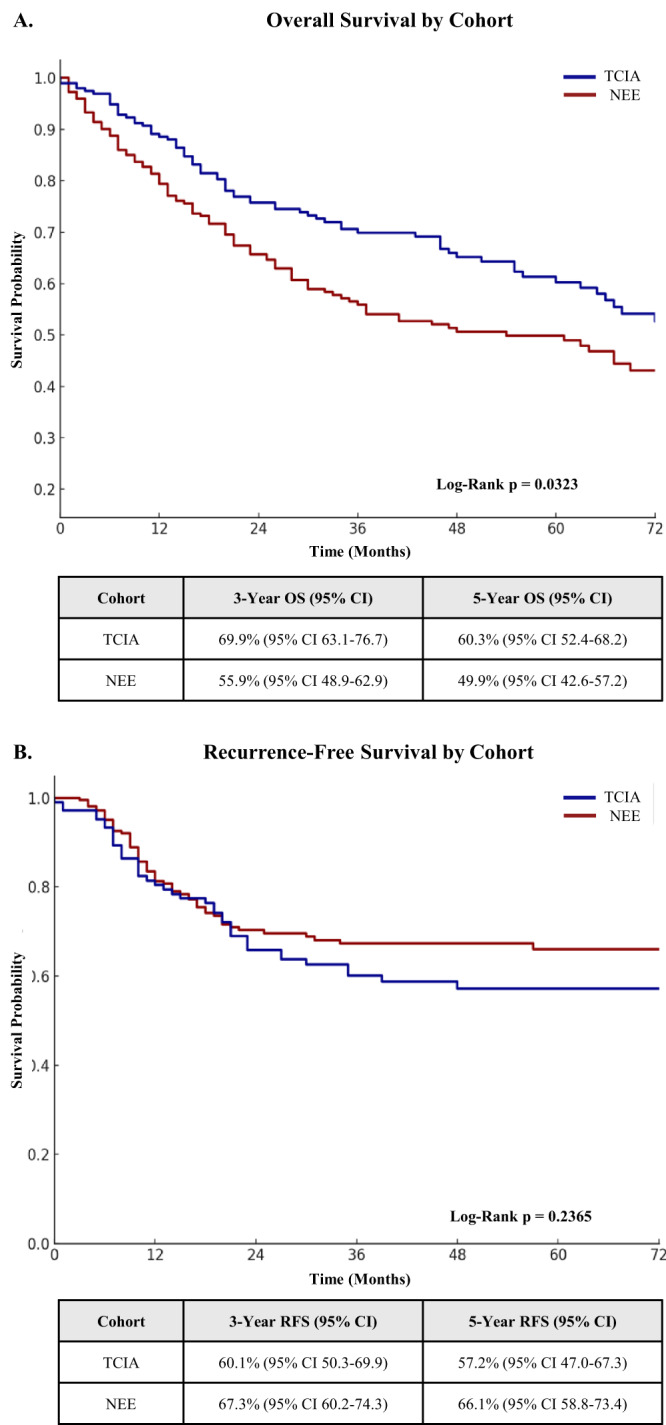
Overall (A) and recurrence-free survival (B) in the North-East England (NEE) and The Cancer Imaging Archive (TCIA) cohorts. In the TCIA cohort, 60.3 per cent of patients were alive at five years versus 49.9 per cent in the NEE cohort. This difference was statistically significant (*p* = 0.0323). There was no significant difference in recurrence-free survival between the cohorts at five years (*p* = 0.2365).

Five-year OS was higher in the TCIA cohort, where 60.3 per cent of patients were alive at five years (95 per cent CI 52.4 to 68.2), compared to 49.9 per cent in the NEE cohort (95 per cent CI 42.6 to 57.2; *p* = 0.0323) (Figure 1A). A similar difference was observed at three years, with survival of 69.9 per cent in TCIA (95 per cent CI 63.1 to 76.7) versus 55.9 per cent in NEE (95 per cent CI 48.9 to 62.9). This difference is likely reflective of broader population health factors, including a higher burden of co-morbidities in the NEE region, rather than differences in treatment efficacy alone.

In contrast, RFS was comparable between cohorts at both three and five years. The five-year RFS was 66.1 per cent in the NEE cohort (95 per cent CI 58.8 to 73.4) and 57.2 per cent in the TCIA cohort (95 per cent CI 47.0 to 67.3), with no statistically significant difference (*p* = 0.2365) (Figure 1B). This suggests that both cohorts achieved similar disease control with initial treatment, and that differences in OS are more likely attributable to non–cancer-related factors such as baseline fitness or competing causes of mortality.

**Figure 2.**
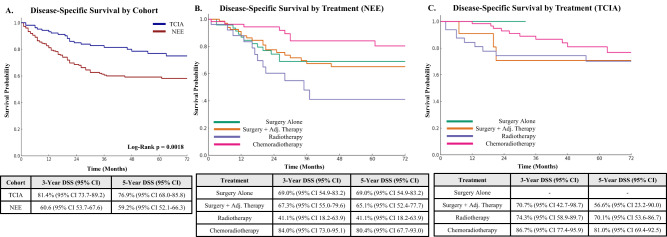
Disease-specific survival (DSS) in the North-East England (NEE) and The Cancer Imaging Archive (TCIA) cohorts. (A) DSS was significantly higher in the TCIA cohort, where 76.9 per cent of patients were alive at five years versus 59.2 per cent in the NEE cohort (p = 0.0018). (B) In the NEE cohort, patients receiving radiotherapy alone had the lowest five-year DSS (41.1 per cent) compared to those treated with surgery and/or chemoradiotherapy. (C) In the TCIA cohort, 5-year DSS exceeded 70 per cent across treatment groups, with the highest in patients receiving chemoradiotherapy (86.7 per cent).

DSS was also higher in the TCIA cohort, with 76.9 per cent of patients alive at five years without disease-related death (95 per cent CI 68.0 to 85.8), compared to 59.2 per cent in the NEE cohort (95 per cent CI 52.1 to 66.3; *p* = 0.0018) (Figure 2A).

When stratified by treatment modality, the most notable difference in DSS was among patients who received radiotherapy alone. In the TCIA cohort, five-year DSS following radiotherapy was 70.1 per cent (95 per cent CI 53.6-86.7), compared to 41.1 per cent in the NEE cohort (95 per cent CI 18.2 to 63.9) (Figure 2C). This may reflect differences in radiotherapy delivery techniques, treatment completion rates, or patient selection for radical intent, particularly within a routine care setting versus a research-focused environment. For other modalities, including chemoradiotherapy and surgery with adjuvant therapy, DSS outcomes were more consistent between cohorts (Figures 2B and 2C), suggesting that multi-modality treatment approaches offer reliable benefit across clinical settings.

## Discussion

We present survival outcomes from two cohorts of patients with advanced laryngeal cancer. The TCIA cohort represents a pooled sample of publicly available research datasets, including data from clinical trials and other institutional studies across multiple high-income countries. In contrast, the NEE cohort reflects real-world outcomes from a single regional centre in the UK. This study offers important insights into the applicability and limitations of research-derived data when translated into routine clinical practice.

At a glance, DSS outcomes were higher in the TCIA cohort compared to the NEE cohort. These differences reflect the issue of selective inclusion. TCIA datasets, derived largely from clinical trials or controlled institutional studies, tend to enrol patients with better performance status, fewer co-morbidities and greater likelihood of completing curative treatment. In many cases, such datasets originate from research environments with selective entry criteria.^9^^–^^11^ By contrast, the NEE cohort represents all consecutive advanced-stage referrals for MDT discussion with initial curative consideration. This inevitably included patients subsequently recognised as having very limited prospects of cure, but who nonetheless form part of the real-world case-mix. Additional factors such as co-morbidity burden, functional limitations, patient preference and institutional protocols also influence outcomes in routine practice.

The NEE cohort included patients who were managed with supportive care alone; however, these individuals were excluded from survival analyses to ensure a consistent comparison across curative-intent treatment modalities. Their inclusion in the broader cohort nonetheless reflects the real-world complexity of managing patients who may be unfit for intensive therapy. Among those included in the survival analysis, radiotherapy alone was often used as a less-intensive curative option, typically selected for patients with significant co-morbidities or limited functional reserve. While treatment selection may contribute to outcome differences, the lower DSS observed in the NEE cohort is more plausibly linked to the underlying health of the population. This region is known for high rates of smoking, greater social deprivation and poorer baseline physical health, all of which likely contribute to reduced treatment tolerance, increased cancer progression and poorer long-term survival.^15^^,^^16^ The North-South health inequality divide remains present in cancer survival data, with worse outcomes attributed to a complex interplay of factors including poor health literacy, delayed first presentation and subsequent late diagnosis.^27^ Although the TCIA cohort has a high degree of missing lifestyle data, limiting direct comparison and real-world applicability, the high percentage of smoking history (95 per cent) in the NEE cohort may contribute to worse DSS.

These disparities highlight the limitations of generalising findings from clinical trials to heterogeneous patient populations, particularly those with varying co-morbidities, functional status and social contexts. While randomised controlled trials (RCTs) remain the gold standard for evaluating treatment efficacy, their results may not fully reflect outcomes in broader, less-selected populations. Real-world data are increasingly recognised as essential for contextualising research findings and guiding decision-making, especially in settings where RCTs are unrepresentative or not feasible.^13^ At the clinical level, both trial and real-world evidence should be considered during informed consent discussions, particularly when outcome disparities are expected.^12^ On a broader scale, modified trial designs that incorporate real-world elements are gaining traction, and regulatory bodies such as the UK Medicines and Healthcare products Regulatory Agency (MHRA) have issued guidance supporting this approach.^28^

This study is limited by its retrospective design and the relatively small sample sizes in both cohorts. The TCIA dataset contains substantial missing data for key lifestyle factors such as smoking and alcohol history, which limits the ability to perform meaningful direct comparisons. In contrast, the NEE dataset is more complete in this regard but represents a single regional population, which may limit the generalisability of its findings. These challenges reflect the inherent difficulties in comparing research datasets with observational real-world data, as discussed above.

This study raises the possibility of a foundational inequality embedded within the structure of clinical research. The significantly poorer outcomes observed in the real-world cohort, despite similar recurrence rates, suggest that trial-based evidence may not adequately represent patients facing socioeconomic deprivation, co-morbidity burden and limited treatment access. These disparities are not incidental but stem from how clinical trials are designed, often excluding complex patients in favour of homogeneity and protocol compliance. As such, our findings imply that outcome gaps may be, in part, driven by systemic underrepresentation. Future research and trial frameworks must actively address this imbalance through broader inclusion criteria, real-world validation arms and policies that prioritise equity to ensure that evidence truly reflects the populations it aims to serve.

- Survival outcomes from clinical trials may not reflect those in real-world populations
- Patients with advanced laryngeal cancer are a heterogeneous group, often affected by co-morbidities and sociodemographic challenges
- In this retrospective analysis, a regional cohort from North-East England demonstrated a higher burden of co-morbidity, distinct treatment patterns and poorer survival compared to research datasets available on The Cancer Imaging Archive (TCIA)
- Future studies should integrate real-world data to better understand population-specific factors and guide more equitable treatment strategies

## Conclusion

While research datasets such as those in TCIA remain essential for evaluating treatment efficacy, they do not fully capture real-world outcomes. The survival disparities and treatment patterns observed in a tertiary head and neck centre in NEE highlight the impact of underlying population health, lifestyle factors and treatment selection in routine clinical care. These findings underscore the importance of assessing the applicability of research-derived data to real-world settings and of recognising the influence of population-specific factors. Future studies should actively incorporate real-world data to complement research findings and better inform both clinical decision-making and health policy.
